# Validation of the adolescent binge eating disorder measure (ADO-BED) among transgender youth and young adults

**DOI:** 10.1186/s40337-023-00816-w

**Published:** 2023-06-07

**Authors:** Whitney Linsenmeyer, Dana Stiles, Sarah Garwood, Andrea Giedinghagen, Christopher Lewis, Gretta Strand

**Affiliations:** 1 Saint Louis University, 3437 Caroline Street, St. Louis, MO 63104 USA; 2 Washington University School of Medicine in St. Louis, 660 South Euclid Ave, St. Louis, MO 63110 USA

**Keywords:** Binge eating disorder, Eating disorder, Transgender, Screening

## Abstract

**Background:**

Transgender youth and young adults are at increased risk for eating disorders, including binge eating disorder, yet few measures have been validated for screening purposes with the transgender population.

**Methods:**

The purpose of this study was to provide initial evidence for the internal consistency and convergent validity of the Adolescent Binge Eating Disorder questionnaire (ADO-BED) in a sample of transgender youth and young adults. 208 participants completed the ADO-BED as part of a routine nutrition screening protocol at a gender center. Exploratory factor analysis and confirmatory factor analysis was used to establish the factor structure of the ADO-BED. Relationships between the ADO-BED, Sick, Control, One Stone, Fat, Food (SCOFF), Nine Item Avoidant/restrictive Intake Disorder (NIAS), Patient Health Questionnaire 9 (PHQ-9), Generalized Anxiety Disorder 7 (GAD-7), and demographic characteristics were explored.

**Results:**

Analyses revealed a one-factor structure of the ADO-BED with good fit to the data in the present sample. The ADO-BED was shown to be significantly related to all convergent validity variables, except the NIAS.

**Conclusions:**

The ADO-BED is a valid measure to screen for BED among transgender youth and young adults. Healthcare professionals can screen all transgender patients for BED, regardless of body size, in order to effectively identify and manage binge eating concerns.

## Abstract (plain language summary)

Transgender individuals are at increased risk for eating disorders, including binge eating disorder. Different questionnaires are used to screen people for eating disorders to know if they need additional evaluation. However, few of the existing questionnaires commonly used to screen for eating disorders have been tested with the transgender population. Therefore, the purpose of this study was to determine if one questionnaire, the Adolescent Binge Eating Disorder questionnaire (ADO-BED), is an accurate measure to screen for binge eating disorder among transgender youth and young adults. The results of this study supported that the ADO-BED is indeed a valid measure to use with transgender patients.

## Background

Eating disorders disproportionately impact the transgender and gender expansive population compared to the cisgender population [1, 2]. Estimates of prevalence range from 2 to 35% of transgender youth and young adults [1, 3]. Theorized rationale for disordered eating among the transgender population include: a desire to attain or suppress body features that align with one’s authentic gender identity; menstrual suppression; pubertal suppression; and a coping mechanism for psychological distress related to minority stress, discrimination and victimization. [1–6]

Emerging research has provided greater specificity regarding prevalence of specific types of eating disorders and related behaviors, including binge eating disorder (BED). Among a clinical sample of youth and young adults presenting to a Midwestern gender clinic, 9% screened positive for BED using the Adolescent Binge Eating Disorder questionnaire (ADO-BED) [6]. Among adolescents at a Seattle-based gender clinic, 26% reported binge eating using selected questions from the Eating Disorder Examination Questionnaire (EDE-Q) [7]. Within a national sample of sexual and gender minority adults, 10% of transgender men, 14% of transgender women, and 12% of gender expansive participants reported binge eating in the past month [8].

Prevalence estimates of diagnosed BED are notably lower than estimates of reported binge eating. Ferrucci and colleagues reported that less than 1% of transgender patients within a medical claims database were diagnosed with BED, though this population had health insurance and access to gender-affirming care [9]. Schvey and colleagues estimated that 2.6% of sexual and gender minority children had BED, a rate three times higher than their non-sexual and gender minority peers. 10]

To address the elevated risk for eating disorders, scholars have underpinned the need to screen transgender patients for eating disorders. However, implementation of this recommendation is limited by the lack of measures validated with transgender populations [1–6]. Validation of eating disorder questionnaires among transgender populations is needed to inform accurate and culturally appropriate screening practices.

## Study purpose and aims

The purpose of this study was to provide initial evidence for the internal consistency and convergent validity of the ADO-BED in a sample of transgender youth and young adults. The ADO-BED was selected given its utility in specifically screening for BED, rather than broadly screening for eating disorders such as the Eating Attitudes Test (EAT-26) or the Eating Disorder Examination Questionnaires (EDE-Q). Furthermore, the ADO-BED was initially validated with an adolescent population, which aligns with the patient population at the study site and other transgender centers that care primarily for youth.

The study aims were to (1) establish and confirm the factor structure of the ADO-BED and (2) explore convergent and divergent validity of the ADO-BED with commonly used measures of anxiety, depression, and disordered eating, and with body mass index percentile.

## Methods

### Participants and setting

Returning patients at the Washington University Transgender Center at St. Louis Children's Hospital transgender center (n = 208) ages 11–22 completed the ADO-BED as part of a nutrition screening protocol for eating disorders and food insecurity. Data were collected from January 2021-June 2022; details of the nutrition screening protocol have been previously reported [6]. The GAD-7 and PHQ-9 were completed as part of routine mental health screening. Additional data collected from electronic medical records included age, sex assigned at birth, gender identity, weight, and height. The Institutional Review Board of the Washington University Transgender Center at St. Louis Children's Hospital provided a waiver of consent given that the nutrition screening is the standard of care.

#### Measures ADO-BED

The ADO-BED is a brief, ten-item measure used to screen for binge eating disorder among adolescents with obesity. Its original development utilized a sample of 94 adolescents ages 12–18 from Geneva University Hospitals in Geneva, Switzerland. A positive answer to either of the first two questions related to 1) eating when not hungry and 2) loss of control overeating was significantly associated with subclinical or clinical BED status when compared to a clinical interview. For scoring purposes, the authors of the ADO-BED identified a high risk of subclinical and clinical BED among participants who responded positively (yes) to questions one or two, and had more than 6 positive answers to the eight additional questions [11].

#### Sick, control, one stone, fat, food (SCOFF)

The SCOFF is a five-item measure used to screen for anorexia nervosa and bulimia nervosa. Scores of 0–5 are used to estimate eating disorder risk [12, 13]. The SCOFF was selected given its brevity and therefore utility at the transgender center, as well as its validation among many racially/ethnically and geographically diverse populations [12, 13].

#### Nine item avoidant/restrictive intake disorder (NIAS)

The NIAS is a nine-item measure used to screen for avoidant/restrictive intake disorder (ARFID) among adolescents and adults, including transgender populations. Scores of 0–45 are used to characterize three restrictive eating patterns, including picky eating, poor appetite or limited interest in eating, and fear of eating consequences [14, 15].

#### Generalized anxiety disorder 7 (GAD-7)

The GAD-7 is a seven-item measure used to screen for generalized anxiety disorder. Scores of 0–21 are used to quantify the degree of anxiety from minimal to severe [16].

#### Patient health questionnaire 9 (PHQ-9)

The PHQ-9 is a nine-item measure used to screen for depression. Scores of 1–27 are used to quantify the degree of depression from minimal to severe [17].

#### Body mass index (BMI)

BMI was calculated using clinic-measured height and weight. Among youth ages 12–19, BMI percentile (BMI%) was reported using the boy or girl growth chart consistent with sex assigned at birth if the patient was on puberty blockers, and/or not on hormone therapy (HT). For patients who were on HT, BMI% was reported using both growth charts. Among young adults ages 20–22, BMI was reported and did not require sex-specific interpretation [18].

### Analytic approach

Using SPSS version 29 and R version 4.2.2, an exploratory factor analysis and confirmatory factor analysis were used to establish and confirm the fit of the ADO-BED survey in the current dataset. The exploratory factor analysis was used to establish the factor loadings. Principal components extraction and oblique rotation were used to interpret the factor loadings, as the items were assumed to be related [19]. Decisions regarding the number of factors to extract were based on eigenvalues < 1 [20], and a visual examination of the scree plot. Significant Bartlett’s test of Sphericity and Kaiser–Meyer–Olkin (KMO) statistic > 0.5 were used to evaluate the data and factor loadings > 0.4 were considered acceptable for the inclusion of each item in a factor.

The confirmatory factor analysis was fit with a diagonal weighted least squares estimator (DWLS) using the R lavaan package [21]. DWLS was chosen since the data was measured on a categorical scale and the positive skew of the ADO-BED. Goodness of fit of the model was evaluated by the following criteria: Root Mean Square Error of Approximation (RMSEA) < 0.05, Comparative Fit Index (CFI) > 0.90, and Standardized Root Mean Square Residual (SRMR) < 0.08 [22]

In order to determine convergent validity, zero-order correlations between the ADO-BED and the convergent validity variables were calculated. These variables include the SCOFF, NIAS, PHQ-9, GAD-7, BMI, BMI %based on sex assigned at birth, and BMI%based on gender identity.

The reliability of the ADO-BED was calculated using Cronbach’s alpha.

## Results

**Fig. 1:**
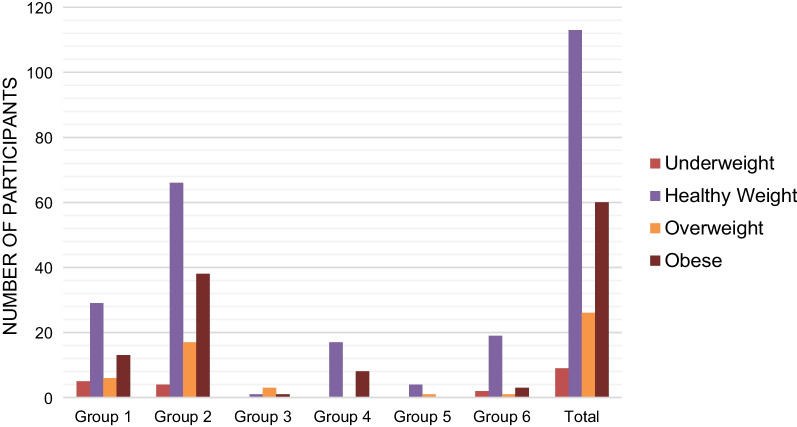
Weight status of the study sample. Group 1: Assigned Male at Birth (AMAB), Puberty Blockers Only and/or No Hormone Therapy. Group 2: Assigned Male at Birth (AMAB), Puberty Blockers Only and/or No Hormone Therapy. Group 3: AMAB, Medical Transition, Growth Chart Consistent with Sex. Group 4: AFAB, Medical Transition, Growth Chart Consistent with Sex. Group 5: AMAB, Medical Transition, Growth Chart of Opposite Sex (Female). Group 6: AFAB, Medical Transition, Growth Chart of Opposite Sex (Male). Total: All Participants, Growth Chart Consistent with Sex

The sample of participants (n = 208) was 11–22 years old with an average age of 15.43 years (SD = 1.86). Participants were assigned female at birth (72.6%) or assigned male at birth (27.4%). Regarding gender identity, participants were transgender female/transfeminine (25.5%), transgender male/transmasculine (55.0%), nonbinary (16.8%), agender (1.4%), or other terms (1.4%); some participants reported multiple gender identity terms, therefore the percentages exceeded 100%. Using BMI or BMI% and the growth chart consistent with sex assigned at birth, the study sample was 4.3% underweight, 54.4% healthy weight, 12.5% overweight, and 28.8% obese (Fig. 1).

Using the scoring criteria of the ADO-BED authors, 13.5% of participants in this study screened positive for clinical or subclinical BED. Slightly more participants (14.4%) responded positively to the question regarding compensatory behaviors (“When you are in these situations do you sometimes need to take action to eliminate what you have just eating [exercise, skip the next meal, self-induce vomiting…]) [11].

The structure of the ADO-BED within the transgender adolescent sample was explored using a principal components factor analysis with oblique rotation. This revealed a one-factor structure with eigenvalues of > 1, which explained 60.13% of the variance. The data appeared to be well suited for factor analysis as indicated by the Kaiser–Meyer–Olkin statistic (0.92) and the significant Bartlett’s Test of Sphericity, X^2^ (45) = 1195.66, p < 0.001 [23–25]. Factor loadings on individual questions were satisfactory: Q1 = 0.856, Q2 = 0.759, Q3 = 0.759, Q4 = 0.712, Q5 = 0.842, Q6 = 0.865, Q7 = 0.834, Q8 = 0.760, Q9 = 0.658, and Q10 = 0.675.

Following the EFA, a confirmatory factor analysis was used for the 1-factor model. Goodness-of-fit-indices supported the one-factor model, which showed good fit to the data: non-significant chi-square, RMSEA = 0.00 [0.00, 0.00], CFI = 1.00, SRMR = 0.054.

See Table 1 for zero-order correlations between the SCOFF and convergent validity variables. There was a significant moderate correlation between the ADO-BED and the SCOFF, and small but significant correlations between the ADO-BED and the PHQ-9, the GAD 7, BMI, BMI% based on sex assigned at birth, and BMI% based on gender identity. The ADO-BED was not significantly related to the NIAS. Reliability was also assessed using Cronbach’s alpha coefficient, which was 0.926.

**Table 1:** Convergent validity correlations (zero-order)

Variable	M (SD)	1	2	3	4	5	6	7
1. ADO-BED total	2.54 (3.24)							
2. SCOFF Total	0.75 (1.09)	.472**	–					
3. NIAS Total	14.22 (9.50)	.118	.409**	–				
4. BMI	25.32 (8.09)	.390**	.246**	.018	–			
5. BMI Percentile (sex)	66.74 (31.09)	.357**	.192**	.006	.722**	–		
6. BMI Percentile (gender)	65.53 (31.51)	.341**	.164**	− .012	.711**	.950**	–	
7. PHQ-9	9.41 (6.05)	.287**	.342**	.363**	.084	.058	.036	–
8. GAD-7	9.14 (6.26)	.302**	.296**	.368**	.115	.156*	.153*	.716**

^*^p < .05, **p < .01. *ADO-BED* adolescent binge eating disorder; *SCOFF* sick, control, one stone, fat, food; *NIAS* nine-item avoidant/restrictive food intake disorder screen; *BMI* body mass index; *BMI percentile (sex)* body mass index percentile for sex assigned at birth; BMI percentile (gender): body mass index percentile for gender identity; *PHQ-9* patient health questionnaire 9; *GAD-7* generalized anxiety disorder 7. BMI was calculated using clinic-measured height and weight. Among youth ages 12–19, BMI% was also reported using the boy or girl growth chart consistent with sex assigned at birth if the patient was on puberty blockers, and/or not on hormone therapy. For patients who were on HT, BMI% was reported using both growth charts. Among young adults ages 20–22, BMI was reported and did not require sex-specific interpretation

## Discussion

BED is often underdiagnosed by healthcare professionals relative to other eating disorders, in part due to relatively new diagnostic criteria and limited screening tools [26, 27]. Other validated screening tools specific to BED include the Binge Eating Scale (BES), the Binge Eating Disorder Screener (BEDS-7), and the Children’s Binge Eating Disorder Scale (C-BEDS) [28–30]. However, these measures have not been validated with the transgender population.

The study findings support that the ADO-BED is a valid measure to screen for BED among transgender youth and young adults ages 11–22 at any weight status. The positive correlation between the ADO-BED and the SCOFF confirms similarities between these measures, such as loss of control over eating or eating under uncomfortably full [11, 12]. The positive correlation between the ADO-BED, BMI and BMI% confirms that patients with BED may have overweight or obesity, though BED can manifest in patients without obesity [31]. A notable proportion (13.5%) of the study sample screened positive for clinical or subclinical BED, and is therefore worthwhile to routinely screen.

Given that the ADO-BED only screens for BED, healthcare professionals can use this measure in tandem with other measures to screen for eating and feeding disorders including anorexia nervosa, bulimia nervosa, and avoidant/restrictive food intake disorder. A notable proportion of participants responded positively to the question regarding compensatory behaviors, which distinguishes BED from bulimia nervosa. Routine screening at gender centers or other facilities that routinely care for the lesbian, gay, bisexual, transgender and queer (LGBTQ) population can increase the likelihood of detecting and managing eating concerns [6].

The results of this study supported that ADO-BED scores were positively correlated with BMI and BMI%. However, all patients should be screened for eating disorders given that BED manifests in populations with and without obesity and often causes significant distress independent of the impact on body weight [31]. Although the ADO-BED was originally validated with adolescents with obesity, this study sample included underweight, healthy weight, overweight and obese participants. Healthcare providers can prepare for how positive ADO-BED screens will be addressed. This may include: Further evaluation of eating patterns and weight history; evaluation for comorbidities such as diabetes and dyslipidemia; and referral to medical, mental health, and allied health professionals trained in gender-affirming care.

## Strengths, limitations and future research

Given that many studies report eating disorder-related data based on a binary view of gender [26], a strength of this study was the gender diverse sample with participants who identified as transgender male, transgender female, non-binary, and genderqueer, among others. This study was limited by data collection at a single site; responses may have varied at other geographical regions within and outside of the United States. Further research can better estimate BED prevalence, especially among patients without obesity, and explore culturally appropriate prevention and treatment approaches.

## Conclusions

The ADO-BED is a valid measure to screen for BED among transgender youth and young adults ages 11–22 at any weight status. Healthcare professionals can screen all transgender patients for BED, regardless of body size, in order to effectively identify and manage binge eating concerns.

## Data Availability

The datasets generated and/or analyzed during the current study are not publicly available due to HIPAA protections but are available from the corresponding author on reasonable request.

## Abbreviations

ADO-BED: Adolescent binge eating disorder questionnaire
SCOFF: Sick, control, one stone, fat, food
NIAS: Nine item avoidant/restrictive intake disorder
PHQ-9: Patient health questionnaire 9
GAD-7: Generalized anxiety disorder 7
BED: Binge eating disorder
EDE-Q: Eating disorder examination questionnaire
BMI: Body mass index
BMI%: BMI percentile
HT: Hormone therapy
KMO: Kaiser–Meyer–Olkin
DWLS: Diagonal weighted least squares estimator
RMSEA: Root mean square error of approximation
CFI: Comparative fit index
SRMR: Standardized root mean square residual
BES: Binge eating scale
BEDS-7: Binge eating disorder screener
C-BEDS: Children’s binge eating disorder scale
LGBTQ: Lesbian, gay, bisexual, transgender and queer
